# Quarantine and Yellow Fever

**Published:** 1855-09

**Authors:** 


### QUARANTINE AND YELLOW FEVER.

Our friend, Dr. Luke P. Blackburn, believed that he had succeeded in keeping yellow fever out of Natchez, two years ago, by quarantine. We had a conversation with him on the subject a few months since, and he was then fully persuaded of the practicability of limiting the spread of this pestilence. The latest intelligence from Natchez is to the effect that yellow fever is prevailing there. What is the matter? Has the vigilance of our friend not been equal to the task of excluding boats and travelers from the city? We are much interested in this matter and shall wait with impatience to learn the explanation of the origin of yellow fever in Natchez. We are sorry to learn that the disease is spreading to many of the towns along the Mississippi river. In Memphis some cases are reported to have originated, but there have been no indications of an epidemic in that place. Norfolk and Portsmouth, in Virginia, are the towns which have suffered most severely from the epidemic this season. It is gratifying to learn that it is at last subsiding in those places. In New Orleans it has not assumed this time its most malignant form and at the last dates was said to be declining. We fear that the experience of our times is not likely to reverse the decision long since passed upon the utility of quarantine regulations.

